# Advancing integrated paediatric care in Australian general practices: Qualitative insights from the SC4C GP-paediatrician model of care

**DOI:** 10.1371/journal.pone.0302815

**Published:** 2024-05-21

**Authors:** Carmen Crespo-Gonzalez, Michael Hodgins, Yvonne Zurynski, Tammy Meyers Morris, Jane Le, Karen Wheeler, Sonia Khano, Stephanie Germano, Harriet Hiscock, Raghu Lingam

**Affiliations:** 1 Population Child Health Research Group, University of New South Wales, Sydney, NSW, Australia; 2 Australian Institute of Health Innovation, Macquarie University, Sydney, NSW, Australia; 3 Sydney Local Health District, Sydney, NSW, Australia; 4 Murdoch Children’s Research Institute, Health Services and Economics Group, Parkville, Victoria, Australia; 5 Central and Eastern Sydney Primary Health Network, Sydney, NSW, Australia; 6 North Western Melbourne Primary Health Network, Parkville, Victoria, Australia; 7 Professorial Fellow, Department of Paediatrics, The University of Melbourne; 8 Sydney Children’s Hospitals Network, Sydney, New South Wales, Australia; University of Melbourne, AUSTRALIA

## Abstract

The Strengthening Care for Children (SC4C) is a general practitioner (GP)-paediatrician integrated model of care that consists of co-consulting sessions and case discussions in the general practice setting, with email and telephone support provided by paediatricians to GPs during weekdays. This model was implemented in 21 general practices in Australia (11 Victoria and 10 New South Wales). Our study aimed to identify the factors moderating the implementation of SC4C from the perspectives of GPs, general practice personnel, paediatricians and families. We conducted a qualitative study as part of the mixed-methods implementation evaluation of the SC4C trial. We collected data through virtual and in-person focus groups at the general practices and phone, virtual and in-person interviews. Data was analysed using an iterative hybrid inductive-deductive thematic analysis. Twenty-one focus groups and thirty-seven interviews were conducted. Overall, participants found SC4C acceptable and suitable for general practices, with GPs willing to learn and expand their paediatric care role. GPs cited improved confidence and knowledge due to the model. Paediatricians reported an enhanced understanding of the general practice context and the strain under which GPs work. GPs and paediatricians reported that this model allowed them to build trust-based relationships with a common goal of improving care for children. Additionally, they felt some aspects, including the lack of remuneration and the work and effort required to deliver the model, need to be considered for the long-term success of the model. Families expressed their satisfaction with the shared knowledge and quality of care jointly delivered by GPs and paediatricians and highlighted that this model of care provides easy access to specialty services without out-of-pocket costs. Future research should focus on finding strategies to ensure the long-term Implementation of this model of care with a particular focus on the individual stressors in general practices.

## Background

Paediatric healthcare systems in many high-income countries are reaching a critical breaking point due to unmet demand for care [1,2]. Remarkable progress has been made in reducing child mortality rates over the past century due to advancements in public health and intensive paediatric care [3]. However, this progress has been accompanied by a rise in chronic illnesses and disabilities [4]. Consequently, the demand for hospital-based care has surged, leading to prolonged waiting times for specialised treatment and escalating healthcare costs [5]. These challenges pose significant strains on the healthcare infrastructure, necessitating strategic interventions to ensure effective and sustainable paediatric healthcare delivery.

An estimated 4.8 million children lived in Australia in 2022, and it is projected that the number of children will reach 6.8 million by 2048 [6]. These children are often referred to allied health or specialist care services. However, waiting times for these services can be lengthy, ranging from weeks to years, both in the public and private sectors for many conditions [7–12]. In 2022, 1.3 million referred paediatric specialist attendances were processed for non-hospital consultations subsidised by the Australian government [13]. Currently, the demand for specialty services has not ceased with some paediatric clinics unable to accommodate new patients with diverse health conditions [8]. In addition, recent data shows that children aged 0–4 years (6% of the population) have the highest rates of presentations to hospital emergency departments (ED) [14]. Some of this increase in demand for ED services can be associated with patients whose conditions are non-urgent or treatable in primary care settings [15–17]. Overcrowding in paediatric EDs has had concerning implications, including a decline in the quality of care provided and a rise in non-elective admissions [5,18].

International data suggests that the existing models of paediatric care in high-income countries are insufficient and unsustainable [19–21]. To address this issue, models of integrated care have emerged as potential solutions [22]. Integrated care is an approach aimed at fortifying people-centric health systems by promoting the comprehensive delivery of quality services throughout a person’s life, tailored to the population’s multidimensional needs and individual requirements [23]. This approach involves a coordinated, multidisciplinary team of healthcare providers collaborating across various care settings and levels [23]. Proposed as a potential means to provide high-quality, patient-centred care in the community, integrated care aims to decrease the reliance on costly hospital-based care, through models, pathways and coordination to support the delivery of person-centred care in the right setting, at the right time and at the right cost [23]. An example of such a model is Strengthening Care for Children (SC4C), an Australian healthcare model collaboratively designed with paediatricians and general practitioners (GPs) in Victoria [24]. This model is adapted from the UK model—Connecting Care for Children (CC4C) [25], which seeks to enhance the quality of care for children through coordination and collaboration among healthcare professionals, ultimately leading to improved health outcomes for children.

### The strengthening care for children model

The SC4C model consists of regular, shared GP-paediatrician 30-minute co-consulting sessions (i.e., GP-led consultations targeted at GPs professional development and shared-care approach) and monthly case discussions (i.e., paediatric education meetings to discuss clinical cases and best practice guidelines) held at the general practice clinic. Additionally, email and telephone support was provided by specialist paediatricians to GPs during weekdays. Following a successful pilot study in 2018 with five Victorian general practices, the SC4C model reduced GP referrals to hospital services, improved family trust in the GP and improved GP confidence in providing paediatric care [26]. These findings led to a translational stepped wedge randomised controlled trial of the model through metropolitan Melbourne and Sydney between 2021–2023 (registration12620001299998) [27,28]. The trial commenced in March 2021 and was implemented in 21 general practices, 11 in Victoria and 10 in New South Wales. Pre-implementation control period data collection (care as usual) began in May 2021 with the first pair of practices implementing the SC4C model in June 2021 [28]. The 12-month intervention period concluded in May 2022 for the first pair of practices and in March 2023 for the last pair. Sustainability data was collected over an 18-month period after the intervention to examine the enduring effects of the SC4C model [28].

During the SC4C trial, paediatricians were co-located at each general practice for 12 months, for one half-day per week for the first 6 months, then reducing to fortnightly. This co-location included scheduled appointments (i.e., co-consultations and case discussions) and non-scheduled conversations to respond to GPs’ queries. The role of the paediatricians was to provide support and advice to GPs to manage paediatric patients and they did not directly assume the role of paediatricians in patients’ follow-up care [28]. Paediatricians were remunerated to perform this duty on a part-time basis and each general practice received AUD $7,000 for their participation. As no Medicare items exist for GP-paediatrician co-consultation sessions, GPs billed using standard Medicare item numbers according to the duration of the co-consultation (Medicare is Australia’s universal health insurance scheme that subsidised a list of professional services with unique item numbers).

An Implementation Evaluation (IE) was conducted as part of the SC4C trial to identify the local contextual differences and approaches to delivering the intervention [27,28]. Evaluating the implementation of complex interventions such as SC4C is crucial to understanding the success or failure of an intervention in a specific context by identifying factors that might moderate its implementation [29,30]. Such evaluations inform intervention adaptations to different contexts and support the adoption of successful models of care at scale. Despite the increasing body of evidence about the effectiveness of integrated primary care interventions, there is limited evidence about the acceptability, appropriateness and adoption as experienced by GPs, paediatricians, families/carers, and other general practice team members (e.g., practice managers, admin staff). Our evaluation of the implementation of SC4C aimed to fill this important gap in the literature. Through the evaluation, we intended to capture valuable insights at the general practice, GP, patient and family level to gain a deep understanding of what worked for whom and in which contexts. This qualitative exploration aimed to provide insight into factors that support and/or hinder the implementation of the SC4C model in primary care settings to inform strategies for optimising implementation at scale.

## Methods

### Study design

We conducted a qualitative study as part of the mixed-methods implementation evaluation of the SC4C trial [27]. We have followed the reporting standards recommended by the Consolidated Criteria for Reporting Qualitative Research (COREQ) [31]. The Consolidated Framework for Implementation Research (CFIR) domains and a logic model informed the development of the interview guides for the semi-structured interviews and focus groups [27].

#### Setting

The implementation evaluation sample included 21 general practice clinics (11 in Victoria, 10 in New South Wales) participating in the SC4C trial within the North Western Melbourne Primary Health Network (NWMPHN) and the Central and Eastern Sydney Primary Health Network (CESPHN) catchment areas.

#### Participant selection

We used purposive sampling to recruit diverse providers and families/carers engaged with SC4C. Once the SC4C trial started, we invited by email all GPs and general practice personnel including practice managers, nurse practitioners, and administrative personnel to participate in a focus group discussion to explore their perceptions and experiences of SC4C six months into the intervention. Additionally, we informed GPs and general practice personnel about the opportunity of participating in a one-on-one interview with the IE researchers during and after the intervention concluded at 12 months. Participant paediatricians were not involved in focus groups but were also invited to participate in a one-on-one interview.

We recruited families/carers whose children participated in a co-consultation with a GP and a paediatrician during the trial survey intervention period by including an item seeking permission to contact them to participate in a qualitative interview about their experience. Those families willing to participate have the option to provide their details at the end of the survey. The IE researchers contacted them via phone and email to schedule this interview.

Participants recruitment was conducted between the 15^th^ of December of 2021 and the 17^th^ of February of 2023.

#### Data collection

We collected data through virtual and in-person focus groups at the general practices and phone, virtual and in-person interviews. The IE researchers CCG (PhD, Research Associate, UNSW, Australia, Female) and MH (PhD, Senior Research Associate, UNSW, Australia, Male) conducted the focus groups and interviews. Both interviewers have more than five years of experience and training in qualitative research. The project managers and research assistants provided potential participants with the aims of the focus and interviews and a brief overview of the IE researchers’ professional background, interests in the area and expertise prior to their consent via email. Additionally, all participants received a participant information sheet and a consent form to sign prior to the interview. None of the participants had any preexisting relationship with the IE researchers conducting the interviews and focus groups.

We explored individuals’ knowledge and perspectives about the model of care; perceived advantages of the model of care; GP and paediatrician self-efficacy to provide care for children; barriers and facilitators; the appropriateness and acceptability of the intervention; and strategies for future implementation [32]. We designed and adapted the individual interview guides according to the type of participants (S1 Appendix). Data collection and analysis of the interviews continued until data saturation was reached [33]. We audio recorded all the interviews and focus groups. The audio recordings were transcribed verbatim by OTTER©, a secure transcription software that turns voice conversations into smart notes and keeps conversations private and only accessible to the person who owns the account. All transcripts were downloaded and deleted from OTTER© and stored in a secure drive. All participant interview audio recordings were listened to test the precision of the transcript and deleted upon completion of the study [34].

### Data analysis

We analysed the data using an iterative hybrid inductive-deductive thematic analysis approach to explore factors (i.e., barriers and enablers) moderating the implementation of SC4C in practice [34,35]. All transcripts were coded and analysed in NVivo Version 12©. The analysis follows five key stages as outlined by Braun et al. [34]: (i) data familiarisation, (ii) generation of initial codes, (iii) searching for themes, (iv) grouping and reviewing themes, and (v) defining and naming themes. The transcripts were read and openly coded by the IE researcher (CCG) [35]. Another researcher (MH) reviewed the initial codes, which were updated following a discussion with the IE research team (MH, RL, YZ). After the initial coding process, themes were generated and discussed with the research team to create the whole data image, including the relationships among themes. Emerging themes were then discussed again with the entire research team (HH, RL, YZ, TM, JL, SK, KW, SG, MH, CCG). We sent a RedCap link to a form with our preliminary summary of the results to all participants in the focus groups and interviews and gave them the opportunity to provide anonymous feedback on the findings.

### Ethics consent and permission

This study is approved by the human research ethics committees of The Royal Children’s Hospital (HREC 65955) and The Sydney Children’s Hospital Network (STE03927). All participants provided written consent. Families/carers who participated in interviews received a $20 gift card for their time.

## Results

Twenty-one focus groups were conducted at 6 months after the intervention started with 94 participants (45 in New South Wales and 49 in Victoria) from 21 GP practices, including 82 GPs, 7 practice managers and 5 nursing and administration staff. The duration of the focus groups was 20–46 minutes. Focus groups were held with groups ranging from 3 to 8 people. Thirty-seven interviews were undertaken with 16 GPs, 5 paediatricians, 3 practice managers and 13 mothers at the end intervention period. At this point, thematic saturation of the main themes was achieved, with no new themes emerging from the data, and therefore no further sampling was conducted. The interviews lasted between 14–50 minutes. Nine participants (8 GPs, 1 PM) completed the findings feedback form.

In the narrative summary of the results below, we discuss the experiences of GPs (GP), paediatricians (P), practice managers (PM) and families (F) participating in this model of care and highlight any contrasting views. Words added to the quotes by the researchers, to clarify meaning, are contained in square brackets. The findings are presented in sections according to the fives themes resulting from our thematic analysis including:

1. The acceptability of the SC4C model: participants’ understanding and attitudes towards the model of care and its various components.
2. Physical versus virtual collaboration between GPs and paediatricians: the influence of different modes of collaboration on the relationships between GPs and paediatricians and the delivery of the model.
3. Practice culture: the influence of general practices preexisting organisational structure on GPs’ involvement with the SC4C model.
4. SC4C increasing and sustaining learning and confidence in primary care: the benefits of implementing the SC4C model.
5. Potential barriers moderating the success and sustainability of SC4C: factors to consider for the long-term success of the model.

### Theme 1. The acceptability of SC4C according to GPs, paediatricians, and families

The model of care and its different components (i.e., co-consultations, case discussions, phone and email support) was overall well received by the participating GPs, paediatricians and families. However, the initial implementation of the model was moderated by GPs’ understanding of the intervention. Supporting quotes can be found in Table 1.

**Table 1:** The acceptability of SC4C to GPs, paediatricians, and families-Theme 1.

Theme	Subthemes		CIFR 2022 Constructs	Quotations
Acceptability	**Individuals’ Attitude**		**Individuals domain:** Model addressed the GP recognised **need** i.e., deficit of confidence and ability in paediatric care.	*GP2*: *“[It was a] good experience to have the paediatrician to keep us updated and also give us the confidence to deal with kids*.*”* *GP47*: *“I feel it’s been really beneficial having the paediatrician sitting in our rooms*. *We are just learning so much more*. *Otherwise*, *we usually refer up to a paediatrician and receive some sort of correspondence back in the form of a letter*.*”* *P2*: *“I think the greatest strength and enjoyment of the project comes from that link*, *breaking down that barrier between hospital and GP”*. *F4*: *"I mentioned this to my mother’s group*, *and everyone was like that is so amazing [in terms of access to paediatricians] and to care*. *It’s something that not many people have*, *but there’s obviously a big need for it*.*"*
	**Intervention Coherence**	Early adoption	**Innovation domain:** GPs and paediatricians’ views on the model **design and complexity**	*GP60*: *“We were one of the first practices that implemented the model*, *so I understand this was a new experience even for the project team and I think this had some sort of impact on how things were explained at the beginning of the project”*. *GP23*: *“I think at first we did not fully understand the whole model but once we started running the model and become familiar with the different components it was not difficult to implement it*.*”*
		Co-consults		*P4*: *“Some of them were just naturally inclined to the project and understood it*, *and they’d sit down and run the consult and we do this really collaborative kind of approach*. *Others would just sit there or would want to put me in their chair*.*”*
		Case discussions		*GP35*: *“She gives all the instructions*, “These are the bulk billing services available and these are the list of places you can refer them*”*, *Those things*, *really helped*. *So*, *with her case discussions*, *we can ask a question if we have something we are not sure about*.* *.* *. *So*, *it’s really helped us with that*.*”* *GP56*: *“Similar to my experience in paediatrics as a resident*, *but it’s much more contextualized for different practice settings*. *So*, *it’s been really useful to get that kind of more general practice-oriented content really helped build my knowledge and skills in that area so far*.*”*
		Phone and email support		*GP12*: *“We didn’t use much of the phone or email mainly because [Paediatrician] was coming like initially*, *like every day*, *there was not much like emergencies for us to phone her*. *Mostly I will wait until she comes”*. *P1*: *“They’ve (referring to highly engaged General Practices) kind of booked everything out*, *and plus corridor conversations and because of that*, *they haven’t uptake the phone and email as much*.*”* *GP [Feedback form]*: *“Rather than having a paediatrician on site*, *better phone or email access to support GPs would suffice and a pathway of referral through this will help*.*”*

#### Individuals’ attitude

GPs had a positive attitude towards working alongside the paediatrician to reinforce and keep them updated in the clinical management of children. Prior to the model GPs often felt left “out of the loop” after referring children to a specialist, citing lack of communication and feedback about their patients after they are discharged from specialist services. SC4C provided an opportunity for GPs to obtain immediate answers to queries about paediatric care and to obtain timely comprehensive information regarding the treatment of their paediatric patients after the co-consultation. Most GPs also commented on families’ attitudes towards the SC4C model and their receptiveness to seeing the specialist during the co-consultation, which provided families a sense of reassurance in the care they were receiving. Overall, families across the sites expressed their satisfaction with the service received. Families felt involved and able to ask questions during these co-consultations, enhancing their confidence in the care received for their child. Paediatricians’ overall impression towards the SC4C model of care was that it reduced the “gap” between hospitals specialists and GPs.

#### Intervention coherence

While the SC4C model was reportedly well integrated within participating general practices, there were some instances in which practices desired more clarity around the logistics of the model and the expectations placed on practice staff. More often than not, practices eventually became more familiar with the processes and expectations of SC4C over time. Additionally, practices whose implementation started later in the stepped-wedge trial reported a smoother implementation experience, largely due to the increased experience of the study team to communicate expectations effectively. Participants reported variation in their experience with the implementation of the model and its different components.

Most GPs understood that the paediatrician’s role during the co-consultations was to support and provide guidance to enhance GPs’ capacity to provide care to children. However, paediatricians reported variability in how GPs approached these co-consultations. In most cases, GPs tended to discuss the cases with the paediatrician before entering the consultation room so they could come to agreement on how to manage the child’s condition. However, some GPs, according to paediatricians, were not as prepared as others, using a more passive approach allowing the paediatrician to guide the consultation while sitting back observing and taking notes.

Case discussions were designed to provide paediatric education on clinical cases and best practice guidelines resources and information. GPs understood the objective and got familiar with the format of the case discussions. The case discussions served as an opportunity for gathering the general practice staff, regardless of their enrolment and engagement with the delivery SC4C model of care, to share knowledge and experiences. Paediatricians reported overall high attendance at these educational sessions and mentioned they tried to adjust them according to the needs of those attending.

Phone and email support was included as a component of the model of care to provide additional support and advice outside the consultation and case discussions time. Although GPs were aware that they could access this support, some GPs reported being unsure how to use this resource. The uptake of this component of the model of care was variable. GPs felt that phone support was not particularly needed while the paediatrician was regularly visiting their practices. However, many GPs cited phone and email support as a resource to use for ongoing support in the future. This was reinforced by the feedback obtained from the findings of our study from a few GPs who felt that just having phone and email support would be equally valuable.

### Theme 2. Physical versus vs virtual collaboration between GPs and paediatricians

SC4C was designed as an in-person integrated model of care, involving GPs and paediatrician collaborative efforts at GP practices. In response to the COVID-19 pandemic, the implementation of the model was largely moved online using video telehealth, thus impeding the direct face-to-face collaboration between GPs and paediatricians. As a consequence of this adaptation, participants strongly felt that the physical components of the model held a greater potency in fostering cooperation and sustaining the model (Table 2).

**Table 2:** Physical versus virtual collaboration between GPs and paediatricians-Theme 2.

Theme	Subthemes	CIFR 2022 constructs	Quotations
**Physical vs virtual collaboration**	**Close collaboration-enhanced understanding**	**Inner setting domain:** GP-Paediatricians **relational connections** and **communication**	*P2*: *“Yeah*, *I think we definitely appreciate the GP culture more in terms of what their workload is like and what are the barriers for them to get proper advice*, *which we know about*, *but we don’t see it until you’re there [referring to their visits to the general practices]*.*”* *GP16*: *"Having the paediatrician in our practice and seeing how she approaches and communicates with the children and their families gave me a better understanding of how they work and what the differences are compared to how we manage children [at the general practice]*.*”*
	**Trust-based relationships-Rapport building (“Corridor conversations”)**		*GP21*: *“Just having casual conversations with her in the corridor*, *if you have any doubts*, *it is fantastic”* *GP46*: *“I didn’t have a co-consult*, *but I did speak with her about specific cases*, *And I felt like you guys weren’t necessarily saying that was worth as much as doing the co-consults*, *but I actually think it is”*. *GP6*: *“That kind of reinforcement or reassurance by the paediatrician that actually what you have advised is absolutely correct and you both just carry on with that decision*, *that’s kind of helpful to reinforce the relationship of the parents with us”*.
	**Telehealth**	**Inner setting domain:** The **compatibility** and **appropriateness** of the virtual delivery of the SC4C model with usual paediatric care at general practices	*GP56*: *“Telehealth*, *I think it’s just really difficult to implement and it wasn’t really effective… a [physical] examination is an important part of paediatrics*, *as well a lot of the time parents feel more reassured having this conversation face-to-face*.*”* *GP33*: *“The challenge in our practice during COVID was that we were limiting and at some stage*, *we didn’t have any patients coming in*, *and some of the parents who wanted to have the co-consultation really wanted to have it face-to-face instead*.*"*

#### Close collaboration/enhanced understanding

Overall, GPs expressed their satisfaction with having SC4C as a collaborative model enabling the provision of care for children alongside paediatricians. This close collaboration allowed paediatricians to better understand the differences between hospital and primary care and helped to close the gap in collaboration between practitioners in these settings. GPs and paediatricians commented that the physical presence of paediatricians in GP practices was key to building engagement and collaboration. Some paediatricians commented that they felt like the in-person representative of hospitals, enhancing the collaboration across primary and tertiary care.

#### Trust-based relationships/rapport building

Having this direct contact with the paediatrician facilitated the establishment of trusting relationships. GPs reported that more informal “corridor conversations” in their practices regarding their paediatric cases as one of the most valuable components of SC4C. Families also reported rapport and relationship between the GPs and paediatricians was evident during the co-consultations. Some GPs resented the feeling that they were merely a gateway along a referral pathway to a specialist rather than the solution for the needs of the family at the beginning of model implementation. However, the physical presence of the paediatrician onsite helped foster rapport and trust, and enabled GPs to understand the value of their role and feel part of the process when caring for children.

#### Telehealth

In response to the COVID-19 pandemic, general practice shifted their model of care to provide care via telehealth as the risks of the pandemic hindered the provision of face-to-face co-consultations. Participant GPs reported that telehealth co-consultations were challenging during the pandemic due to the difficulties conducting physical examinations and a preference for face-to-face communication with paediatric patients and their families.

### Theme 3. Practice culture enabling or limiting SC4C success

While SC4C had the potential to enable effective collaboration between primary and specialty care, this collaboration also hinged upon a supportive GP practice culture. Most GPs agreed that the care model was crucial for upskilling their paediatric practice and, most importantly, improving their skills to interact with paediatric patients at the primary care level. Despite GPs’ positive reporting of the model, their engagement in some practices was not as high as expected. This was noted by paediatricians to more likely be attributed to a practice’s organisational culture, rather than an individual GP’s willingness to be part of the project. Supporting quotations related with this theme can be found in Table 3.

**Table 3:** Practice culture-enabling or limiting SC4C success (Theme 3).

Themes	Subthemes	CIFR 2022 constructs	Quotations
**Practice culture**	**Pre-existing teamwork culture**	**Inner setting domain:** GP **relational connections** and **communication** with other GPs.	*P5*: *“The ones that are less engaged*, *I think*, *seem to have a more individualistic approach to practicing medicine at the GP level*. *So*, *they’re all themselves*, *they are their own single entity*, *they go in*, *they do their thing*, *they go out and they go home*.*”* *P2*: *"It would be so much better if GPs developed the habit of once a week*, *meet and discuss- who should we be sending a (child with) speech delays to*, *and who is good in this area*? *And what did you have*? *What happened when you referred that one there*, *and if we could invent that culture that would be amazing*.*"* *P1*: *"I guess part of the barrier [to collaborate] even is that in some of the General Practices*, *there is not even a room where people can actually meet*. *We often ended up like in the kitchen [referring to the case discussion setting]”*.
	**General practice management and stressors**	**Inner setting domain:** The **compatibility** of the SC4C model with the general practices’ workflows and its **relative priority.**	*P3*: *“I think it’s very much dependent on the culture in a given practice and the other stressors that are going on within that practice*. *And thinking about it I think it’s much more dependent on the practice than on its own individuals”*. *P1*: *“The engagement of the General Practices comes down to workplace culture and attitude to be honest*, *which is something that it’s really hard to engage (assess) from a study point of view but that’s definitely impacted on the practice’s performance and also that ties in with the practice managers as well*, *and their relationship to the GPs*.*”*

Paediatricians highlighted several pivotal factors within the practice environment that influenced GPs’ level of involvement. The presence of an existing culture of teamwork and collaboration at GP practices emerged as a crucial element in fostering and solidifying the rapport with GPs, as those physicians were already attuned to the value of cooperative work, sharing insights and experiences, rather than functioning in isolation. Nevertheless, adopting this collaborative stance proved more challenging for certain practices, where the lack of a shared physical space, like a communal area, hindered this approach.

The role of practice managers and their leadership garnered significant attention from paediatricians as a catalyst for GPs’ engagement. Recognising their pivotal position, practice managers were deemed essential in cultivating participation and enhancing the model’s implementation. Additionally, the demands of the practices’ workload and the customary time constraint of 15 minutes per consultation also emerged as factors reducing GPs’ engagement across select practices.

### Theme 4. SC4C increasing and sustaining learning and confidence in primary care

The introduction of the SC4C care model facilitated the growth of GP expertise in paediatric management. Consequently, both medical practitioners and families gained a heightened sense of assurance and confidence in the ability of GPs to effectively care for children (Table 4).

**Table 4:** SC4C increasing and sustaining learning and confidence in primary care-Theme 4.

Themes	Subthemes	CIFR 2022 constructs	Quotations
**SC4C increasing and sustaining learning and confidence in primary care**	**Learning and professional development**	**Inner setting domain:** Collaborating with the paediatrician provides GPs continuous **access to knowledge and information** to upskill and successfully deliver the model.	*GP19*: *“Just watching her do examinations*, *just watching the way she interacts with children*. *It’s always good to learn*.*”* *GP88*: *“I have learnt magnificent things but some of the behavioural things and developmental things*. *I feel like I’ve got a few more skills and more confidence to be able to do things”*. *F3*: *“It is really reassuring that your GP is getting regular access to that kind of professional development*. *It is really fantastic*.*”* *F7*: *“I think it’s great for the GPs to get that experience and to get that knowledge in the room as well*.*”* *PM7*: *“Great*, *really spectacular*, *we’ve learned so much*. *All the doctors have been approachable and happy to change*. *There have been children with issues on the day*, *and they popped in the room*, *had a conversation*, *and tried to sort things out*, *which is very valuable*.*”*
		**Innovation outcomes domain:** The **impact** of implementing SC4C for GPs, general practice staff and families	*P1*: *“I think it’s been a positive change with the project definitely they are cooperating*, *and you do see a difference in terms of changing their management and changing and improving their paediatric care”*. *P2*: *“More than reducing the number of referrals*, *I think they are now more aware of the services and resources that are available for them*.*”* *GP34*: *“I do see the benefits of the model for us*, *but I still believe that there is not much difference for the patients in this area*. *Because at the end of the day they will still need to wait for a really long period to see a specialist”*. *GP [feedback form]*: *“I would still not feel confident managing these specialised conditions even after the SC4C program*. *However*, *I feel more informed about these conditions and better able to suggest other management options to the patients*.*”*
	**Confidence building**	**Innovation outcomes domain:** The **impact** that delivering the model has on GPs confidence in paediatric care.	*F4*: *“I know GPs will walk away from this experience with an added level of perspective*. *So*, *I do feel more comfortable in that respect*, *but for certain conditions I will still prefer to visit the paediatrician”*. *GP78*: *“The Paediatrician has increased my confidence that you are still making a difference*. *Because they [families]*, *perhaps they don’t have that sort of stability in life apart from having a relationship with a doctor*.*”* *GP25*: *“Sometimes people [referring to GPs] become uncomfortable that they will be exposed by being vulnerable in front of somebody and saying*, *“I don’t know this”*. *I don’t see anything bad because you can’t know everything*. *But some people are not comfortable with the idea of maybe being judged for what they know*, *or what they do not know”*.
	**Access to quality care**	**Individuals domain:** Model addressed the family and GPs stated **need** (i.e., access to quality care.)	*F6*: *“It was really fantastic that you could get that access to care*. *If there was any way to get this model implemented for longer time*, *I would be happy to pay an extra fee for the service*.*”* *F8*: *“It was amazing to get that specialist knowledge on top of your GP and with your GP*. *I just think it was one of the best experiences of access to quality healthcare*, *to be honest*.*”*
	**Perceived need**	**Individuals domains:** GPs’ **motivation** to implement the SC4C model based on their **needs**.	*GP44*: *“We didn’t actually get that many co-consults each week for the paediatrician*. *There were times when she was just sort of sitting there doing nothing much*. *Maybe that’s because our GPs*, *we’re relatively familiar with looking after children and didn’t feel so much the need or I’m not sure*.*”* *GP73*: *“I was confident to manage children before this model*. *However*, *having her in here*, *have provide that extra reassurance that it is going to be really beneficial moving forward*.*”* *GP10*: *“I have a few co-consults but for me personally*, *it was hard to get patients to come back*. *Yeah*, *they just weren’t interested*, *they’re busy and they’re coming to see you*.”

#### Learning and professional development

Learning and reinforcement of essential skills, including how to interact and communicate with children and their families, examinations, early detection of behavioural and developmental concerns, and referral pathways, among others, were appreciated by GPs. A few GPs felt the model was more beneficial for their learning and professional development rather than for their patients. This perception arose from the fact that while paediatricians offered guidance and support during co-consultations, they were not directly involved in subsequent patient follow-up care. As a result, paediatric patients still required referrals to other specialists post-consultation.

Regarding the GPs’ referral patterns, both GPs and paediatricians agree this experience would probably not necessarily reduce the number of referrals but would improve the quality of those referrals instead. Paediatricians reported GPs referrals, in most instances, were justified. Thus, the paediatrician’s role was to assist them in providing more comprehensive information regarding those referrals. Additionally, paediatricians felt their role in SC4C has been helpful in raising GPs’ awareness of available resources for children that families can access while waiting to see a specialist. GPs supported this by saying they now have additional resources to provide families with additional information and advice. On the feedback survey, a GP commented that while still needing more confidence in managing specialised conditions, the SC4C model was useful for becoming more informed about those conditions and gaining knowledge regarding other options to manage their paediatric patients. GPs and PMs commented on the benefits this model of care offers to their patients allowing them to obtain support and immediate answers to their questions. Some families also highlighted the importance of professional development of their GPs as part of this model of care, which improved their confidence and reassurance in their GP’s skills to manage their children.

#### Confidence building

Most families cited that they had built rapport and trusted their GPs before this model of care. However, some families still reported that they would prefer to visit a paediatrician for their child’s condition in the future. From the GPs’ perspectives, they valued and cited improvements in their confidence because of participating in this model care. GPs’ perceived capability and knowledge to manage paediatric patients influenced their participation in SC4C. Some GPs expressed feeling vulnerable to being exposed to the “expert opinion” of paediatricians. In some instances, feeling afraid to be judged was reported as a motive negatively influencing the enrolment or engagement of some GPs in SC4C. Nevertheless, most GPs expressed that the paediatricians’ interpersonal skills were crucial to making them feel comfortable and able to ask questions.

#### Access to quality care

The accessibility to specialists provided by SC4C was overwhelmingly noted as a key benefit by GPs and families. The long waiting times to visit a private paediatrician was a significant problem that families across both states dealt with. In more affluent versus poorer areas with greater availability and access to private paediatricians, participants still reported a preference to have access to co-consultations in the long term due to the improved access to quality care with the expertise of both GPs and paediatricians.

From the perspective of families, having direct access to a specialist without paying extra fees was an incentive for them to participate in the co-consultation. For this reason, they expressed they would like to have access to this service in the future even if the continuation of the service means for them to pay an extra fee.

#### Perceived need

Those GPs that were less likely to engage with the model cited a familiarity with managing children, not seeing the need to involve the paediatrician. Most GPs expressed they would like to have the paediatrician onsite for a more extended period. However, they still felt they could manage their paediatric patients, and having the paediatrician there has reinforced their self-efficacy in the area. While most GPs cited positive family attitudes, some GPs mentioned family unwillingness to attend a follow-up consultation with the SC4C paediatrician when their needs had already been met in a consultation with their GP, or they preferred to visit their own private paediatrician.

### Theme 5. Potential barriers moderating the future success and sustainability of SC4C

While there are noticeable benefits from implementing the model of care, participant GPs and paediatricians felt there were some aspects including the lack of remuneration and the work and effort required to deliver the model that need to be considered for the long-term success of the model (Table 5).

**Table 5:** Potential barriers moderating the future success and sustainability of SC4C -Theme 5.

Themes	Subthemes	CIFR 2022 constructs	Quotations
**Potential barriers**	**Financial burden**	**Inner setting domain:** The availability of **funding** support to implement and deliver the SC4C model	*GP4*: *"So in most of my co-consult*, *I just do Medicare billing*. *Therefore*, *the billing dropped because of that*. *But to me*, *that is not important*. *Because the learning from it is much more valuable than the money*.*”* *GP32*: *"[Referring to the number of GPs enrolled in the practice] everybody works differently*, *unfortunately*, *due to poor Medicare funding*, *some of the people rely on seeing more patients per hour*.*"*
	**Perceived effort**	**Individuals’ subdomain:** Paediatricians and GP personnel **motivation, opportunity, and capability** to successfully implement and deliver the SC4C model. **Innovation domain:** The **adaptability** of the SC4C model to GPs’ and general practices’ needs.	*PM1*: *“There was some sort of pushing to fill up the spaces and the books*. *Honestly*, *there was no time to fill the books*, *because we were just launching*, *we didn’t have any patients booked for the following Monday*.*”* *P2*: *“The paediatricians on this project have to be flexible and adaptive to the different styles which I think really increases the success of the project because if you have someone that is a bit more rigid*, *and a GP that’s a little bit more rigid it’s a little bit harder to be collaborative*.*”* *GP9*: *“Well*, *the first couple of weeks of the co-consult*, *I didn’t know what was going to be like*, *so I think it was a bit hard to coordinate my time*, *with the paediatrician’s time”*. *P5*: *“A program like this is very dependent on the energy that the paediatricians put into it*, *that was just reflective of the sheer amount of driving we were doing and then*, *when we get there*, *there is a huge amount of energy that we put in in terms of engaging those GPs*.*”* *P4*:*”Now it is taking less of a toll on me*, *because I’ve got less practices on board*, *and I’ve absolutely engaged those GPs so that initial work that we had to do has started to pay off and that has become easier to do*.*”*

#### Financial burden

General practices are under considerable strain due to the workload, staffing levels and different billing models relying on the number of patients seen per hour. Therefore, the implementation of this model of care can be seen as an additional pressure for them. While each general practice was paid for their participation in the model of care, GPs in this study did not directly receive any financial incentives, so they relied on their usual source of income. When asked about the financial impact of delivering the model of care, most participants cited no significant changes in their revenue, however some cited a drop in their ability to bill effectively. While potentially costly, GPs felt that the shared experience and the learning component of the model offset the potential drawback in cost.

#### Perceived effort

The paperwork and administrative burden to set up the SC4C model hindered initial implementation. In the first practices in which SC4C was implemented, practice managers expressed feeling overwhelmed about the amount of correspondence received from the research team. While not cited as an issue by all practice managers and GPs, this was an issue that proved a pivotal challenge for some practices, reducing their engagement with the study team and model. Adapting the SC4C model to the different circumstances of the general practices was highlighted to ensure effective scale up of SC4C. Some GPs expressed feeling a bit “pressured” by the formality of “having to reach” specific numbers of booked co-consultations. There were also difficulties around aligning paediatrics visits while GPs were onsite and scheduling the one-hour case discussions between GPs and paediatricians due to the high workload of GPs.

From paediatricians’ perspectives, the most significant burden was the time spent driving between practices and the number of practices (up to five) they visited at some points during the implementation. Paediatricians felt the SC4C model was highly dependent on their energy to engage and motivate GPs, which was an additional challenge when they had to visit several practices at once. As a suggestion for the future, they recommended visiting several practices located closer together, or reduce the number of practices to visit per paediatrician.

## Discussion

This is one of the first qualitative studies to analyse the implementation of an integrated care model delivered to children. The findings of our qualitative analysis demonstrate the acceptability of the SC4C model among GPs, paediatricians and families as well as general practice personnel. Participating GPs reported enhanced confidence and knowledge as a direct result of their involvement in this model. Paediatricians, on the other hand, noted the model’s reliance on their energy and effort to engage and motivate GPs. Their participation in the model provided valuable insights into the functioning of general practices and the challenges faced by GPs. Establishing trusting relationships and working collaboratively towards the common goal of improving paediatric care emerged as a significant and highly valued aspect of the model, as highlighted by both GPs and paediatricians. Notably, feedback from families revealed a high level of satisfaction with the shared knowledge and quality of care provided by the participating doctors. Families also emphasised the crucial role played by the SC4C model in facilitating easy access to specialty services without lengthy waiting times or additional costs.

While SC4C was found to be a suitable model for general practices, the initial adoption and integration of the model within routine practice was challenging in some instances. A study by Palinkas et al. assessing barriers and facilitators of the Safe Environment model for children, also identified the disruption in the practice workflow and the additional work involved as barrier to the early implementation of the model [36]. To address these challenges and improve the feasibility and adoption of an integrated model of care, adequate planning is crucial, including initial discussions with personnel involved. These discussions should focus on clarifying the scope of the model and establishing clear expectations for providers. By involving all relevant stakeholders from the beginning, potential issues can be identified and addressed early on, improving the chances of successful implementation [37].

The face-to-face contact of this integrated model of care was one of the aspects reported as most beneficial for both GPs and paediatricians. In this context, shared care is highly reliant on the capacity of healthcare providers to share their knowledge and experiences, which requires high levels of interaction and collaboration [38]. One of the most crucial aspects to consider in enhancing this collaboration is establishing clear communication between stakeholders. Having in-person contact with collaborators allows for a closer relationship and rapport with the added benefit of obtaining answers and feedback in real-time [39]. Relational literature in healthcare has suggested that a shared sense of identity and belonging, resulting in collaborative processes (e.g., conversation, joint problem-solving activities), creates a rapport that enhances cooperative behaviours and motivation [40]. However, an increasing body of evidence reports the benefits of telehealth consultations for patients and healthcare providers, including flexibility and comfort of seeing the doctor from home [41,42]. Therefore, a future hybrid model of integrated care that involves both face-to-face and telehealth features should be considered. A hybrid version of SC4C would allow GPs and families to have equity access to quality care and face-to-face contact with the specialists if needed while allowing the paediatrician to engage with more practices as travel time to practices would be reduced.

Paediatricians reported that general practices’ culture and infrastructure greatly influenced the engagement of general practices and GPs with the model’s components. These findings echo those from a pilot study of an integrated care model for children with autism and mental health needs, reporting that the implementation of integrated model is not a “one-size-fits-all” approach and likely will vary based on organisational and patient-level characteristics [43]. Recent research has also reported that implementing behavioural change initiatives and integrated care models in general practices is often moderated by GPs’ attitudes, which are highly dependent on their workload, time pressure, stress levels, and remuneration models [44–46]. To achieve service improvement, there is a need to promote an organisational culture that focuses on enhancing collective behaviour for the provision of care [47]. GPs and nurses participating in a quality improvement program combining e-learning and peer group meetings stated they used group meeting to discuss their experiences and to collaborate on design initiatives to improve the routines in the general practice [48]. Therefore, having a whole practice approach with established shared goals and objectives, promoting collaboration, and teamwork seems to be crucial to successful implementation [49].

SC4C model has been found to be key for the professional development of GPs resulting in increased confidence, knowledge and skills to manage children which was noted by both families and paediatricians. A recent trial found that families were more likely to feel more comfortable relying on their GPs and more satisfied with the quality of care received for their children after the GPs received training in mental health [50]. Moreover, parental satisfaction has also been linked to the easy access to quality care for children which still seems to be lacking in Australia [20]. The literature reports two dimensions of quality of care -access (i.e., availability of services and resources) and effectiveness (i.e., received care according to patients’ needs) [51]. While GPs’ lack of remuneration for the provision of co-consultations has not been reported as a barrier for the implementation of the SC4C, this aspect should be carefully considered for the long-term scale up and sustainability of the model of care. Australian GPs source of income is highly dependent on Medicare rebates which have not increased more than 7.3%, from 2017 ($37.5) to 2022 ($39.75) for a standard consultation, despite the raising costs due to the inflation [52,53]. Therefore, it is worthwhile to mention that SC4C seems to fit within the needs and expectations of Australian families and thus, the long-term implementation of this integrated model should be considered by Government and funding agencies.

One key learning obtained from the feedback from GPs and paediatricians has been the importance of adapting the SC4C model and its components to respond to the needs of the setting in which it is implemented. This is in accordance with a recent study that found that the adaptability according to the workflow of the clinics to meet patients’ needs was a positive influencer of integrated hypertension-HIV services [54]. Most importantly, a recent study exploring mechanisms for implementing integrated care programmes for patients with multi-morbidity conditions found that reaching the balance between formal structures and flexibility was one of the key drivers of their implementation. Reaching this flexibility was found to be dependent on previous agreements and ongoing contact between providers [55]. The concept of adaptability has also been linked to "adaptive leadership", referring to the crucial role of those in leadership positions towards staff behaviour and relationship shift to support patient-centred care. This leadership mindset consists of supporting individuals through challenges to thrive in complex and challenging environments [56,57].

## Limitations

Owing to the qualitative nature of our research, the full spectrum of opinions of other GPs, paediatricians and families in Australia might not be represented. However, the opinions expressed by participants was not uniform as highlighted in our analysis. Additionally, a limitation is that our study is metropolitan based and thus, needs further evaluation in rural and remote areas. Another limitation is that our sample was restricted to English-speaking participants, due to financial limitations of the trial. However, with our large qualitative sample size, purposive sampling techniques and data saturation we are confident the outcome of this study reflects an accurate representation of our participants.

## Conclusions

The SC4C model of care has demonstrated acceptability among general practices and GPs seeking to expand their capabilities in the management of paediatric patients. Families have expressed high satisfaction levels owing to the improved accessibility and provision of high-quality care facilitated by the SC4C model. Paediatricians recognised this model as an excellent opportunity to bridge the gap between hospital and primary care, thereby enhancing their understanding and collaboration between sites. However, it is essential to acknowledge that the individual stressors faced by general practices, including workload, leadership, and teamwork, significantly influence the varying levels of engagement with the model observed across different practices. Into the future, key success factors to consider will include effective senior leadership, shared values and additional funding. Subsequent research endeavours should prioritise the identification of strategies that ensure sustained and broad-based implementation of this or similar care models that children and their families are able to access in an equitable manner. Particular attention should be given to the influence of Medicare’s current billing model on Australian general practices and GPs.

## Supporting information

S1 Checklist (PDF)

S1 Appendix SC4C interview guides. (PDF)
