# Supplementary material for: Advancing integrated paediatric care in Australian general practices: Qualitative insights from the SC4C GP-paediatrician model of care
Source: PLoS One. 2024 May 21;19(5):e0302815. doi: 10.1371/journal.pone.0302815 (PMC11108132; doi:10.1371/journal.pone.0302815)
Supplement: S1 Appendix — (PDF) [file pone.0302815.s002.pdf]

SC4C Interview guides

HREC #65955

Introductions

Confirm consent and re-iterate that the interview will be recorded; transcripts will be de-identified and any quotes used will not be attributed by name

Strengthening Care for Children – GP Interview Guide

General

- What do you believe were the incentives or motivations for implementing SC4C in your practice?

Implementation Process

- Tell us about how the implementation of SC4C happened in your practice?
- What is your impression about how practical it was to implement SC4C in your practice?
- How and to what extent did SC4C impact on the usual way your practice works?
- What kinds of infrastructure changes had to be made to accommodate SC4C in your practice? *Prompts: Changes in scope of practice? Changes in formal policies? Changes in information systems or electronic records systems? Other?*
- Were the online resources, marketing materials, and toolkits provided through the SC4C trial helpful in implementing SC4C? In which ways were they helpful or not helpful?
- What additional resources would you have liked when SC4C was being implemented?
- What do you believe could have been done differently to improve the implementation of SC4C in your practice?

Fidelity and unexpected consequences

- When SC4C was first starting up what were your expectations of SC4C?
- Now that SC4C has been implemented for (X months) what were the positive consequences of implementing SC4C for your practice?
- Were there any surprising effects (positive or negative) on your practice because of SC4C?
- Do you believe that implementing SC4C has had financial implications for your practice?
- *If yes*, tell us about these financial implications?
- *Prompts – Did your practice incur additional costs because of loss of clients, changes in workflows, etc…*

Changes in attitudes

- Has implementing SC4C changed your attitudes to providing care to children in your practice? If so in what ways?
- Do you think the attitudes of colleagues in your practice changed because of SC4C? *If so* in what ways?

Sustainability

- When the trial is finished, do you believe that your practice will continue the SC4C model of care or a similar model? *Please provide reasons*.
- What incentives do you think are needed to continue SC4C or a similar model of care for your practice?
- What is your opinion about implementing SC4C in GP contexts different to yours, e.g. a smaller practice or a practice in a rural region?

Impacts on knowledge, confidence and practice

- During the SC4C trial how frequently did you attend co-consults, case discussions, or access email and phone support?
- Out of the co-consults, case discussions, access to paediatricians via email or phone, which were most beneficial to your practice and why?
- From a personal point of view, what has it been like to work alongside paediatricians as part of SC4C?
- What have you learnt from working alongside paediatricians?
- To what extent do you feel you are better able to look after children in primary care because of SC4C?

Strengthening Care for Children – Paediatrician Interview Guide

- What do you believe were the incentives or motivations for implementing SC4C in practice?

Implementation Process

- Tell us about how the implementation of SC4C happened for the practices you attended?
- What is your impression about how practical it was to implement SC4C in these practices?
- How and to what extent did SC4C impact on the usual way you work?

Fidelity and unexpected consequences

- When SC4C was first starting up what were your expectations of SC4C?
- Now that SC4C has been implemented for (X months) what were the positive consequences of implementing SC4C for your practice?
- Were there any surprising effects (positive or negative) on your practice because of SC4C?

Changes in attitudes

- Has implementing SC4C changed your attitudes to providing care to children? If so in what ways?
- Has implementing SC4C changed your attitudes to working with primary care? If so in what ways?

Sustainability

- When the trial is finished, would you like to continue the SC4C model of care or a similar model? *Please provide reasons*.
- What incentives do you think are needed to continue SC4C or a similar model of care for paediatricians generally?
- What is your opinion about implementing SC4C in other GP contexts, e.g. a smaller practice or a practice in a rural region?

Impacts on knowledge, confidence and practice

- During the SC4C trial how frequently did you attend co-consults, case discussions, or access email and phone support?
- Out of the co-consults, case discussions, email or phone support, which do you think were most beneficial to GPs and why?
- Out of the co-consults, case discussions, email or phone support, which do you think were most beneficial to your own knowledge about primary care and why?

- From a personal point of view, what has it been like to work alongside GPs as part of SC4C?
- What have you learnt from working alongside GPs?

To what extent do you feel you are better able to work alongside primary care because of SC4C?

Strengthening Care for Children- Families Interview Guide

1. When you made the appointment, were you expecting a co-consultation with the GP and paediatrician?
2. Describe your experience of the co-consultation with GPs and paediatricians
   a) Tell me about how information and advice was provided to you during the co-consultation.
   b) How did this compare to your previous experiences of seeing only the GP?
3. Do you feel more confident about seeing your GP with a paediatrician present? Why/why not?
4. What did you like/dislike about the co-consultation?
   Prompts: time taken, number of people in the consultation room, usefulness of advice received, was advice given clear?
5. Were you actively involved in making decisions about you/your child's care during the co-consultation?
6. Compared with consultations with GPs only, do you feel you were more or less involved in making decisions about your/your child's care?
7. What do you think are the main benefits of having this co-consult with both doctors?
8. What would you like to see done better next time you attend a co-consultation with a GP and paediatrician?
9. Do you think it's realistic to offer co-consultations with the GP and paediatrician in the long-term and after this trial has finished? Why/why not?
10. Without this study, would you have had any issues accessing care from a paediatrician if your child needed it?
